# Retraction Note: SNPs in genes implicated in radiation response are associated with radiotoxicity and evoke roles as predictive and prognostic biomarkers

**DOI:** 10.1186/s13014-018-1027-9

**Published:** 2018-04-26

**Authors:** Ghazi Alsbeih, Medhat El-Sebaie, Najla Al-Harbi, Khaled Al-Hadyan, Mohamed Shoukri, Nasser Al-Rajhi

**Affiliations:** 1 Radiation Biology Section, Biomedical Physics Department, King Faisal Specialist Hospital and Research Centre, P.O. Box 3354, Riyadh, 11211 Saudi Arabia; 2 Radiation Oncology Section, Oncology Centre, King Faisal Specialist Hospital and Research Centre, P.O. Box 3354, Riyadh, 11211 Saudi Arabia; 3 National Biotechnology Center, King Faisal Specialist Hospital and Research Centre, P.O. Box 3354, Riyadh, 11211 Saudi Arabia; 4 Radiation Biology Section, Biomedical Physics Department, KFSHRC, MBC-03, P.O. Box 3354, Riyadh, 11211 Saudi Arabia

## Retraction

The authors are retracting this article [1] because the data have already been published in [2] making this a redundant publication. Ghazi Alsbeih, Najla Al-Harbi, Khaled Al-Hadyan, Mohamed Shoukri and Nasser Al-Rajhi agree with this retraction. Medhat El-Sebaie did not respond to our correspondence.
